# Visual Dysfunction in Chinese Children With Developmental Dyslexia: Magnocellular-Dorsal Pathway Deficit or Noise Exclusion Deficit?

**DOI:** 10.3389/fpsyg.2020.00958

**Published:** 2020-06-05

**Authors:** Yuzhu Ji, Hong-Yan Bi

**Affiliations:** ^1^CAS Key Laboratory of Behavioral Science, Center for Brain Science and Learning Difficulties, Institute of Psychology, Chinese Academy of Sciences, Beijing, China; ^2^Department of Psychology, University of Chinese Academy of Sciences, Beijing, China

**Keywords:** developmental dyslexia, magnocellular theory, noise exclusion, Chinese children, visual dysfunction

## Abstract

Many studies have suggested that children with developmental dyslexia (DD) not only show phonological deficit but also have difficulties in visual processing, especially in non-alphabetic languages such as Chinese. However, mechanisms underlying this impairment in vision are still unclear. Visual magnocellular deficit theory suggests that the difficulties in the visual processing of dyslexia are caused by the dysfunction of the magnocellular system. However, some researchers have pointed out that previous studies supporting the magnocellular theory did not control for the role of “noise”. The visual processing difficulties of dyslexia might be related to the noise exclusion deficit. The present study aims to examine these two possible explanations via two experiments. In experiment 1, we recruited 26 Chinese children with DD and 26 chronological age–matched controls (CA) from grades 3 to 5. We compared the Gabor contrast sensitivity between the two groups in high-noise and low-noise conditions. Results showed a significant between-group difference in contrast sensitivity in only the high-noise condition. In experiment 2, we recruited another 29 DD and 29 CA and compared the coherent motion/form sensitivity in the high- and low-noise conditions. Results also showed that DD exhibited lower coherent motion and form sensitivities than CA in the high-noise condition, whereas no evidence was observed that the group difference was significant in the low-noise condition. These results suggest that Chinese children with dyslexia have noise exclusion deficit, supporting the noise exclusion hypothesis. The present study provides evidence for revealing the visual dysfunction of dyslexia from the Chinese perspective. The nature of the perceptual noise exclusion and the relationship between the two theoretical hypotheses are discussed.

## Introduction

The main feature of developmental dyslexia (DD) is a specific and significant impairment in the acquisition of reading skills that is not solely accounted for by mental age, visual acuity problems, or inadequate schooling (World Health Organization, 2011). The phonological deficit theory, which is widely accepted in alphabetic languages, postulates that the difficulties in representation, storage, or retrieval of speech sounds have a negative impact on the development of grapheme-phenome correspondences, eventually leading to poor phonological skills and reading disability in dyslexia (Snowling, 2001; Ramus, 2003). However, some researchers believe that the specific reading impairments might be traced to some general perceptual processing problems, such as auditory temporal processing impairment (Tallal, 2004), visual magnocellular deficit (Stein, 1997, 2001, 2014), and cerebellar deficit (Nicolson et al., 2001; Nicolson and Fawcett, 2007).

Initially, DD was described as word-blindness, which emphasized the importance of visual processing problems in addition to the phonological deficit. In the late 19th century, studies reported some general visual deficits in dyslexia (Morgan, 1896; Orton, 1925). Later, more and more studies found it was related to the magnocellular pathway deficit. Dyslexics did poorly in processing the rapid visual information that is carried by the visual magnocellular system and the postmortem study also provided evidence that the magnocellular layers of the lateral geniculate nucleus (LGN) in dyslexia was more variable in shape and smaller in general compared with controls (Livingstone et al., 1991; Galaburda and Livingstone, 1993). Therefore, the magnocellular theory was proposed to explain the visual dysfunction of dyslexia (Stein, 1997, 2001). Some researchers also named it as magnocellular-dorsal theory (e.g., Gori et al., 2014), because the dorsal stream mainly received information from the magnocellular pathway (Livingstone and Hubel, 1988; Boden and Giaschi, 2007). In recent decades, many studies found impaired visual magnocellular-dorsal pathway function in dyslexics by means of behavioral and neuroimaging measurements (Boets et al., 2011; Jednoróg et al., 2011), which confirmed the magnocellular theory. However, Sperling et al. (2005, 2006) pointed out that some previous studies that found magnocellular-dorsal deficits in dyslexics used stimuli with noisy conditions, so they assumed that the visual difficulties in DD might be associated with a noise exclusion deficit rather than magnocellular pathway deficit.

Sperling et al. (2005) first used a Gabor contrast sensitivity task to examine their hypothesis. This paradigm is often used to detect the magnocellular pathway function of dyslexia. In their study, the magnocellular and parvocellular stimuli were presented with or without noisy display. They found that children with dyslexia showed lower contrast sensitivity than controls only in the high-noise condition, no matter which type of stimuli were used. After that, Sperling et al. (2006) used the coherent motion task, which is usually used to detect the dorsal pathway function of dyslexia. They measured the coherent motion sensitivities, respectively, in the high-noise condition, in which the contrast of the signal dots was the same as the noise dots, and the low-noise condition, in which the signal dots were red. Results showed that the perceptual threshold of the coherent motion of dyslexics in the high-noise condition was significantly higher than that of controls, whereas the group difference disappeared in the low-noise condition, suggesting the noise exclusion deficit in dyslexia. Some subsequent studies also supported this hypothesis. Northway et al. (2010) used the symbol discrimination task to measure the contrast sensitivity. Results showed that the contrast sensitivity of DD was lower than that of the control group in the high-noise condition, while no evidence was observed that the group differences were significant in the low-noise condition. Conlon et al. (2012) used the coherent motion task, which included three conditions: (1) low signal contrast with high noise contrast, (2) the same contrasts of signal and noise, and (3) high signal contrast with low noise contrast (Conlon et al., 2012). They found that DD exhibited a higher threshold in conditions except for the low-noise condition.

It seems that deficits in noise exclusion contribute to the etiology of dyslexia, but the studies mentioned above did not take the global form task into account. In previous studies that supported the magnocellular-dorsal theory, the global form task was used as the control condition (non-motion) (Hansen et al., 2001; Conlon et al., 2009). DD showed comparable performance with the control group in this task but exhibited poor coherent motion sensitivity. In the global form task, the contrasts of signal and noise were the same as in the coherent motion condition, which means that the stimuli were also presented in the high-noise condition. If the visual impairments in dyslexia are due to noise rather than motion, it should be observed that DD exhibited poor performance in the high-noise condition not only in the motion task but also in the static task. In addition, in the study by Sperling et al. (2005), the authors did not find the deficit of dyslexia to be specific to the magnocellular stimuli, which was inconsistent with the results of previous studies (e.g., Borsting et al., 1996; Demb et al., 1998; Slaghuis and Ryan, 1999; Kevan and Pammer, 2008). It can be seen that the spatial frequency of Gabor was different in these studies. Early primate studies found that only stimuli with both low spatial frequency [e.g., 1.0 cycles per degree (cpd)] and high temporal frequency (e.g., 10 Hz) were unaffected by the destruction of parvocellular layers but that it induced contrast sensitivity reductions following lesions of magnocellular layers (Merigan and Eskin, 1986; Merigan et al., 1991a, b; Skottun, 2000). It has been proved by functional magnetic resonance imaging studies that the anatomical organization and functional properties of the human LGN showed similar patterns compared with monkey LGN (Schneider et al., 2004; Zhang et al., 2015). However, in Sperling et al.’s (2005), the frequency of magnocellular Gabor was 2 cpd, which might not be completely detected by the magnocellular system.

As compared with alphabetic languages, Chinese as a logographic script has more complex spatial structures without clear grapheme–phoneme corresponding rules. Because of the language specificity, it seems that the deficits of Chinese individuals with dyslexia are different from those with alphabetic dyslexia (Shu et al., 2006; Yang and Bi, 2011; Yang et al., 2013, 2016). Despite the discrepancies, Chinese children with dyslexia also exhibit similar visual processing difficulties. Studies found a lower sensitivity of Chinese DD than typically developing children in the coherent motion task, and the sensitivity was correlated with some reading-related skills such as orthographic awareness, phonological awareness, and picture-naming speed (Meng et al., 2011; Qian and Bi, 2014). Researchers have explained these results as reflecting the magnocellular-dorsal pathway deficit in Chinese children with dyslexia. However, just as Sperling et al. (2005, 2006) indicated, these studies also used high-noise display, so the results could also be explained as the noise exclusion deficit in Chinese dyslexia. It remains unclear whether the visual dysfunction in Chinese children with dyslexia is attributed to the magnocellular-dorsal deficit or the noise exclusion deficit.

The aim of the present study was to examine the two theoretical hypotheses by two experiments in Chinese children with DD. Experiment 1 used a Gabor contrast sensitivity task in which the magnocellular and parvocellular visual stimuli were presented with high and low external noise. Experiment 2 used a coherent motion task and global form task in the high-noise and low-noise conditions. We hypothesized that if DD showed the magnocellular-dorsal deficit, the worse performance of children with dyslexia should be observed in the M condition of the contrast sensitivity task and coherent motion task; if DD showed the noise exclusion deficit, the worse performance of children with dyslexia should be observed in the high-noise conditions whether the stimuli were related to the M condition/motion or not.

## Experiment 1

### Methods

#### Participants

Fifty-two Chinese children in grades 3–5 were recruited from two primary schools in Beijing. Half of them were DD (14 boys; age range: 8.22–11.95 years) and half were chronological age–matched healthy children (CA; 18 boys; age range: 8.24–11.65 years). The screening criteria of DD were a reading ability test score at least 1.5 standard deviations below grade average in the Standardized Character Recognition Test (Wang and Tao, 1993) and IQ greater than 85 as measured by Raven’s Standard Progressive Matrices (Raven et al., 1996). These criteria are widely used in Chinese studies for screening Mandarin-speaking children with dyslexia (e.g., Shu et al., 2006; Wang et al., 2010; Meng et al., 2011; Qian and Bi, 2014). We also measured some reading-related skills for children, including word reading fluency, phonological awareness, morphological awareness, and rapid automatized naming (RAN). Dyslexic children showed significantly worse performance than the controls in all tests except phonological awareness test (marginally significance). It can support the reliability of screening for dyslexia. All participants were right-handed. They had normal hearing and normal or corrected-to-normal vision without any other neurological abnormalities. This study was approved by the ethics committee of the Institute of Psychology, Chinese Academy of Sciences. Detailed information of each group is shown in Table 1.

**TABLE 1:** Characteristics of the two groups in experiment 1 (*M* ± *SD*).

	CA (*n* = 26)	DD (*n* = 26)	*t*	*p*	Cohen’s d
Age (years)	10.25 ± 0.95	10.15 ± 1.05	0.35	0.73	0.10
IQ (standard score)	112.62 ± 12.18	111.04 ± 12.05	0.47	0.64	0.13
Reading ability (number of Chinese characters)	2870.48 ± 540.78	1875.45 ± 531.73	6.70	<0.001	1.86
Word reading fluency (number of correct words/1 min)	107.31 ± 18.92	76.69 ± 17.24	6.10	<0.001	1.69
Phonological awareness (number of correct items)	19.65 ± 5.84	16.73 ± 6.13	1.76	0.085	0.49
Morphological awareness (number of correct items)	25.27 ± 5.19	21.00 ± 5.93	2.76	0.008	0.77
RAN (sec)	14.76 ± 3.05	16.37 ± 2.47	−2.10	0.04	−0.58

#### Measures of Reading-Related Skills

##### Standardized character recognition test

In this test, participants were instructed to write down a compound word with each of the target morpheme characters. Characters are divided into 10 groups based on reading difficulty (206 characters for 3rd graders, 174 characters for 4th graders, and 210 characters for 5th graders). Each correct response was given one point. The score for each group of characters was calculated by multiplying the total points by the corresponding coefficient of difficulty. The final score for each participant was the sum of sub-scores for all 10 character groups to estimate of the number of Chinese characters the children actually recognize.

##### Word reading fluency

This task contains 160 single Chinese characters with high frequency. Children were asked to read all this words as fast as possible in 1 min. The number of the correct answer were the final score of the task.

##### Phonological awareness

In this task, children were orally presented with three syllables of Chinese characters and were asked to judge which syllable was different from the others in initial consonant, vowels or tone (e.g., /meng3/was different from/gao1/and/bao4/in vowels). There were 30 items in total and the final score was the number of correct items.

##### Morphological awareness

In this task, children were presented with one pair of 2-morpheme words which contains the same morpheme (e.g., “
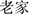
” rat and “
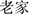
” home). They need to judge if the same morpheme in different words has the same meaning. There were 20 items in total and the final score was the number of correct items.

##### Rapid automatized naming (RAN)

Performance of children’s RAN of pictures and digits were collected. Five pictures (flower, book, dog, hand, and shoes) and five digits (2, 4, 6, 7, and 9) were used, respectively, in the two tasks. Pictures/digits were repeatedly presented visually in random order on a 6 × 5 row-column grid. Participants were asked to name each picture/digit in sequence as quickly as possible. The total naming time was collected. Each task was conducted twice, and the average score was used as the final RAN score.

#### Stimuli and Procedure

**FIGURE 1:**
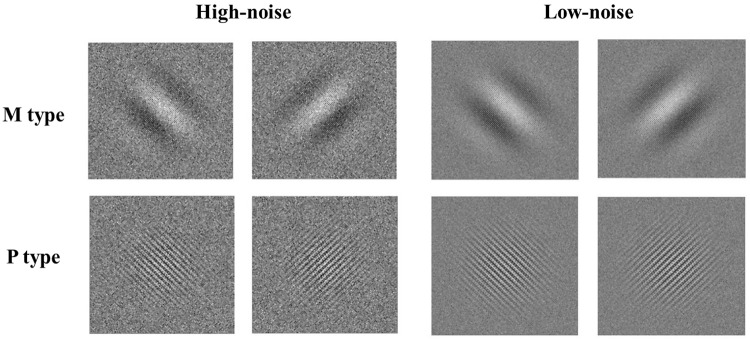
Examples of M and P Gabor with high noise and low noise.

As shown in Figure 1, stimuli consisted of a Gabor pattern of sine wave gratings with checkerboard noise. The magnocellular-type gratings had a spatial frequency of 0.5 cpd and flickered in counter phase at a rate of 15 reversals/s (temporal frequency = 15 Hz). The parvocellular type gratings had a spatial frequency of 5 cpd and did not reverse phase (temporal frequency = 5 Hz). Both of these kinds of gratings had two orientations (45° or 135°). Noise consisted of 2 × 2 pixel patches. The contrast of each pixel patch was sampled from a Gaussian distribution. In the high-noise condition, the contrast of the brightest and darkest pixel patches was 100%; in the low-noise condition, it was 40%, selected by a pilot study to avoid the ceiling effects. Noise also reversed phase when accompanied by M stimuli but was static when accompanied by P stimuli. The size of each kind of stimuli was 6° × 6°.

The task was programmed using Matlab R2015b with Psychtoolbox extensions. The monitor resolution was 1366 × 768, and its vertical refresh rate was 60 Hz. Stimuli were shown on a gray background with a luminance of 51.73 cd/m^2^. Children sat 60 cm from the computer screen and were given the opportunity to practice. In the formal experiment, a fixation was first shown at the center of the screen for 250 ms, and then the stimuli appeared. After 200 ms, a blank screen was presented, and children were asked to judge the orientation of stimuli by pressing the corresponding keys without time limitation. The contrast of Gabor in a single trail was determined by a 3-down/1-up staircase. The initial contrast was 50%. Before the first reversal, a step amounted to change contrast by 20% of the present contrast level. After that, it was changed by 10% of the present level. The program stopped when children reached 150 trials or 10 times of reversal. The average contrast level for the last five reversals was taken to estimate the contrast threshold. Four separated staircases were applied for different conditions, and the order was counterbalanced across participants.

#### Data Analysis

The three-way repeated-measures analysis of variance (ANOVA) was conducted firstly, with a between-subject factor (group: DD, CA) and two within-subject factors (stimulus type: M, P; noisy condition: high noise, low noise). Then the two-way repeated-measures ANOVAs were conducted for the high-noise condition and low-noise condition, respectively, with a between-subject factor (group: DD, CA) and a within-subject factor (stimuli type: M, P).

### Results

The thresholds of M/P Gabor with high noise and low noise in the two groups are presented in Table 2.

**TABLE 2:** Contrast thresholds (%) for different stimuli in DD and CA (*M* ± *SD*).

	High-noise		Low-noise	
		
	M type	P type	M type	P type
CA	11.68 ± 3.50	13.91 ± 3.45	5.1 ± 1.7	5.2 ± 1.9
DD	14.36 ± 3.95	14.89 ± 2.74	5.5 ± 1.6	5.9 ± 2.1

It showed that the three-way interaction was marginally significant (*F*_1_,_50_ = 3.62, *p* = 0.063, partial η^2^ = 0.068). In order to better understand this effect, the two-way ANOVAs were further conducted for the high-noise and low-noise conditions separately.

#### High-Noise Condition

There was a significant main effect of stimuli type (*F*_1_,_50_ = 5.20, *p* = 0.027, partial η^2^ = 0.094), with a higher threshold for P stimuli than M stimuli. The main effect of group was also significant (*F*_1_,_50_ = 6.14, *p* = 0.017, partial η^2^ = 0.109). DD exhibited a higher threshold than CA. The interaction between group and stimuli type was non-significant (*F*_1_,_50_ = 1.99, *p* = 0.165, partial η^2^ = 0.038).

#### Low-Noise Condition

**FIGURE 2:**
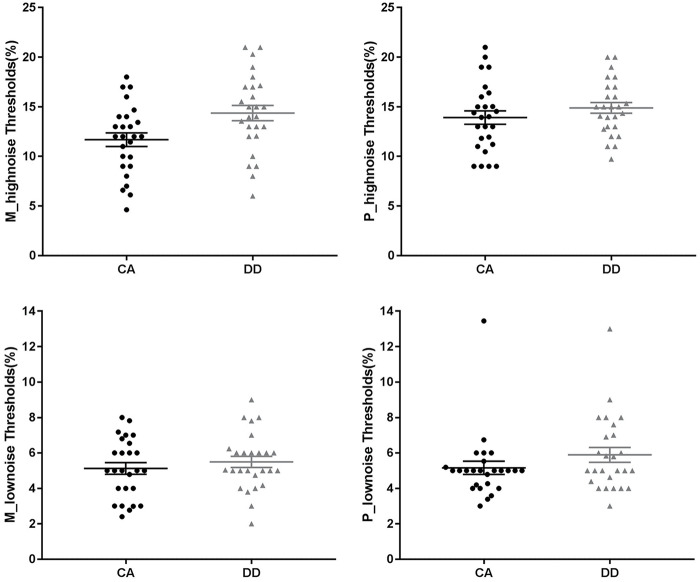
M/P-type Gabor contrast thresholds of two groups in the different noisy conditions. The longest line in the middle denotes the means and the other two lines denote the standard error. CA, chronological age–matched controls; DD, developmental dyslexia.

The ANOVA showed non-significant main effects of group (*F*_1_,_50_ = 2.30, *p* = 0.136, partial η^2^ = 0.044) and stimuli type (*F*_1_,_50_ = 0.36, *p* = 0.553, partial η^2^ = 0.007). The interaction between group and stimuli type was also non-significant (*F*_1_,_50_ = 0.26, *p* = 0.614, partial η^2^ = 0.005). See Figure 2.

### Discussion

In experiment 1, we used the Gabor contrast sensitivity task to investigate the magnocellular/parvocellular pathway function and the role of noise in Chinese children with dyslexia. Results showed that, in only the high-noise condition, dyslexia exhibited significantly lower sensitivities than the control group no matter what type of stimuli they processed. In the low-noise condition, none of the main effects and no interaction was found. These results indicated that Chinese children with dyslexia had noise exclusion deficit, supporting the noise exclusion hypothesis.

Even though more strict parameters were used to set up the magnocellular and parvocellular Gabor as compared with Sperling et al.’s (2005), we still did not find the selective deficit of dyslexia in processing M-type stimuli. This was not in line with expectations and inconsistent with previous studies (Borsting et al., 1996; Demb et al., 1998; Slaghuis and Ryan, 1999; Kevan and Pammer, 2008). The reasons might be as follows. First, this task involved only the spatial frequency and temporal frequency of Gabor to discriminate M-type and P-type stimuli. The information about contrast and color were not taken into account. Actually, magnocellular layers not only preferred higher temporal frequency and lower spatial frequency but were also sensitive to lower contrast and color-blindness; parvocellular layers preferred lower temporal frequency, higher spatial frequency, and higher contrast and showed robust response to both chromatic and achromatic stimuli (Zhang et al., 2015). Second, the neuronal responses in magnocellular layers and parvocellular layers were preferentially rather than exclusively tuned to M-type and P-type stimuli (Skottun, 2015; Zhang et al., 2016). A behavioral experiment cannot directly measure the responses of M layers and P layers to different types of stimuli; thus, it might not be able to detect such a subtle deficit.

## Experiment 2

### Methods

#### Participants

Another 58 Chinese children in grades 3–5 were recruited. Half were DD (21 boys; age range: 8.78–11.51 years) and half were CA (22 boys; age range: 8.63–11.45 years). All participants were right-handed. The screening criteria of DD and CA were same as in experiment 1. We also measured some reading-related skills for children (same as experiment 1). Dyslexic children showed significantly worse performance than the controls in all tests. It can support the reliability of screening for dyslexia. Detailed information about each group is shown in Table 3.

**TABLE 3:** Characteristics of the two groups in experiment 2 (*M* ± *SD*).

	CA (*n* = 29)	DD (*n* = 29)	*t*	*p*	Cohen’s d
Age (years)	10.50 ± 0.85	10.36 ± 0.92	0.60	0.55	0.16
IQ (standard score)	112.07 ± 12.18	108.62 ± 13.43	1.03	0.31	0.27
Reading ability (number of Chinese characters)	2881.64 ± 261.72	1984.26 ± 325.37	11.57	<0.001	3.04
Word reading fluency (number of correct words/1 min)	105.03 ± 18.38	74.24 ± 13.27	7.32	<0.001	1.92
Phonological awareness (number of correct items)	27.24 ± 2.82	24.90 ± 4.84	2.26	0.03	0.59
Morphological awareness (number of correct items)	16.83 ± 1.73	14.93 ± 2.05	3.80	<0.001	1.00
RAN (sec)	13.82 ± 1.88	15.89 ± 2.04	−4.00	<0.001	−1.05

#### Stimuli and Procedure

**FIGURE 3:**
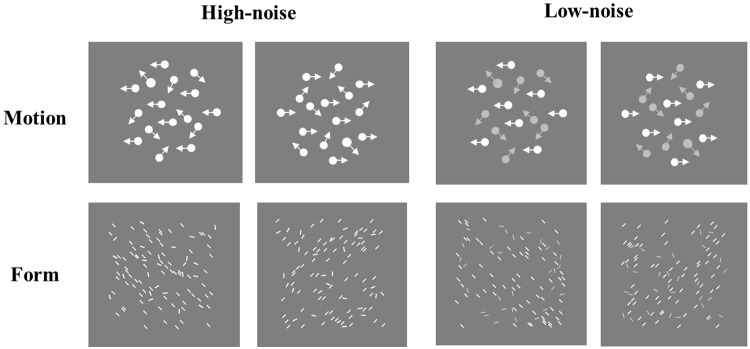
Examples of coherent motion/global form task in the high-noise/low-noise condition. Please note: the contrast, size, and number of dots/lines shown in the two tasks are for illustration.

The coherent motion stimuli were generated by a random-dot kinematogram, which comprised 100 moving white dots with a diameter of 0.14° and a speed of 2°/s. The signal dots moved coherently in a single direction (left or right), and the noise dots moved randomly. To prevent eye tracking, each dot had a lifetime of 3 frames, after which the dot disappeared and was regenerated at a randomly selected location (the radius of moving scope ranged from 1° to 4°). Compared with the coherent motion task, we designed a new global form task, which comprised 100 static lines in a 6° × 6° area. The size of each line was 0.26° × 0.06°. The orientation of signal lines was fixed (45° or 135°) and that of noise lines was random. Both tasks had two versions: high noise and low noise, respectively. In the high-noise condition, the signal contrast was the same as the noise contrast (both were 63.88%); in the low-noise version, the signal contrast was also 63.88%, but the noise contrast was 58.52% (see Figure 3). These contrasts were selected by a pilot study to avoid the ceiling effects in the low-noise condition.

All tasks were also programmed by Matlab R2015b with Psychtoolbox extensions. The monitor resolution was 1366 × 768, and its vertical refresh rate was 60 Hz. Stimuli were shown on a gray background with luminance of 12.98 cd/m^2^. Children sat 60 cm from the computer screen and were given the opportunity to practice. The procedure of the two tasks was quite similar. A fixation was first shown at the center of the screen for 250 ms. Then it disappeared in the global form task but remained on screen through one single trial in the coherent motion task. Stimuli were shown for 1000 ms. After that, a blank screen was presented, and children were asked to judge the direction of the signal dots or the orientation of signal lines by pressing the corresponding keys with no time limitation. The proportion of signals in a single trail was determined by a 3-down/1-up staircase. The initial proportion was 50%. Before the first reversal, a step amounted to the change proportion of signals by 20% of the present proportion level. After that, it was changed by 10% of the present level. The program stopped when children reached 150 trials or 10 times of reversal. The average proportion level for the last five reversals was used to estimate the threshold. Four separated staircases were applied for the two versions of two tasks, and the order was counterbalanced across participants.

#### Data Analysis

The three-way repeated-measures ANOVA was conducted firstly, with a between-subject factor (group: DD, CA) and two within-subject factors (task: motion, form; noisy condition: high noise, low noise). Then the two-way repeated-measures ANOVAs were conducted for the high-noise condition and low-noise condition, respectively, with a between-subject factor (group: DD, CA) and a within-subject factor (task: motion, form).

### Results

The thresholds of coherent motion and global form of the two groups in the high-noise and low-noise conditions are shown in Table 4.

**TABLE 4:** Thresholds (%) for different tasks and conditions in DD and CA (*M* ± *SD*).

	High-noise		Low-noise	
		
	Motion	Form	Motion	Form
CA	17.59 ± 8.93	17.23 ± 6.37	11.00 ± 6.57	12.56 ± 5.84
DD	21.79 ± 11.25	21.44 ± 6.70	11.91 ± 6.49	16.05 ± 7.97

It showed that the three-way interaction was non-significant (*F*_1_,_56_ = 0.69, *p* = 0.410, partial η^2^ = 0.012). The interaction between noise and group did not reach significance (*F*_1_,_56_ = 0.99, *p* = 0.323, partial η^2^ = 0.017), but the interaction between task and noise was significant (*F*_1_,_56_ = 4.29, *p* = 0.043, partial η^2^ = 0.071). We proceed with the two-way ANOVAs separately for the high-noise and low-noise conditions, in order to further understand the results and keep consistent with experiment 1.

#### High-Noise Condition

The ANOVA showed a significant main effect of group (*F*_1_,_56_ = 5.96, *p* = 0.018, partial η^2^ = 0.096), and DD exhibited a higher threshold than CA. Neither the main effect of task (*F*_1_,_56_ = 0.06, *p* = 0.807, partial η^2^ = 0.001) nor the interaction between group and task was significant (*F*_1_,_56_ < 0.001, *p* = 0.996, partial η^2^ < 0.001).

#### Low-Noise Condition

**FIGURE 4:**
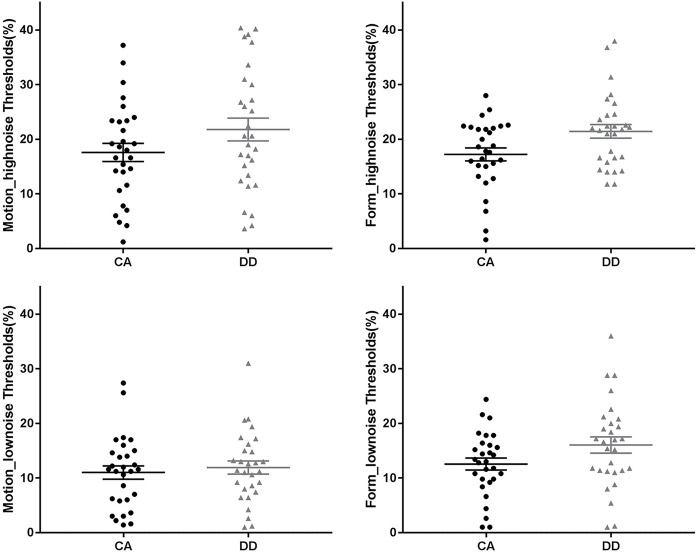
Coherent motion/form thresholds of two groups in the different noisy conditions. The longest line in the middle denotes the means and the other two lines denote the standard error. CA, chronological age–matched controls; DD, developmental dyslexia.

The main effect of task was significant (*F*_1_,_56_ = 9.40, *p* = 0.003, partial η^2^ = 0.144), in that the threshold for the form task was higher than that for the motion task. However, neither the main effects of group (*F*_1_,_56_ = 2.11, *p* = 0.152, partial η^2^ = 0.036) and task nor the interaction (*F*_1_,_56_ = 1.93, *p* = 0.171, partial η^2^ = 0.033) was significant. See Figure 4.

### Discussion

Results of experiment 2 showed that DD exhibited a higher threshold than CA in the high-noise condition, whereas no evidence was observed that the group difference was significant in the low-noise condition. This suggests that Chinese children with dyslexia have noise exclusion deficit, whether it is related to motion or not, also supporting the noise exclusion hypothesis.

One of the main findings in experiment 2 was that Chinese children with dyslexia showed a noise exclusion deficit in the coherent motion task, which is the same as the results from alphabetic languages studies (Sperling et al., 2006; Conlon et al., 2009; Northway et al., 2010). This revealed that the visual difficulties of Chinese DD were related to noise rather than motion, and the noise exclusion deficit in DD might be a cultural-general deficit. In addition, results of experiment 2 also showed a higher threshold of dyslexic children in the global form task with high-noise condition. This result was inconsistent with the previous studies of Hansen et al. (2001), Conlon et al. (2009), and Meng et al. (2011). In their studies, stimuli were presented only in the high-noise condition, and the poor sensitivity of dyslexia was observed in the coherent motion task rather than global form task. The possible reason for the inconsistent results might be the different difficulties of the two tasks. Thresholds of the global form task were higher than that of the coherent motion task observed in those three studies. This might result in a possible floor effect in the global form task, resulting in the inability to find a difference between the two groups. In the present study, no evidence was observed that the task main effect was significant in the high-noise condition, which means that the difficulties of the two tasks were the same. In this case, we found only a significant group main effect, suggesting that Chinese children with dyslexia have poor coherent sensitivities and that this is related to the noise rather than stimuli type.

## General Discussion

The present study examined two theoretical hypotheses to explain the visual dysfunction of Chinese children with dyslexia. Two experiments consistently showed that dyslexic children showed poorer performance than controls only in the high-noise condition no matter what kind of stimuli types and tasks they processed. This suggests that Chinese children with dyslexia have a noise exclusion deficit, supporting the noise exclusion hypothesis. The present study provides evidence for revealing the cognitive mechanism of visual dysfunction in dyslexia from the Chinese perspective.

### Noise Exclusion Deficit in Chinese Children With Dyslexia

This study was based on the two previous studies of Sperling et al. (2005, 2006) and improved the experimental paradigm. In experiment 1, we used strict spatial frequency and temporal frequency for the M Gabor and P Gabor. In experiment 2, we designed a new global form task as a control to the motion task. In that case, the results of the two experiments still showed the noise exclusion deficit of Chinese children with dyslexia, which was consistent with the results of Sperling et al.’s studies in an alphabetic language cultural context. This might suggest that dyslexia have a relatively robust noise exclusion deficit across different language cultures. Despite the discrepancies between different language systems, Chinese children with dyslexia also exhibited the same cognitive mechanism of their visual processing difficulties. Similarly, a previous neuroimaging study also found a common brain activation for semantic decisions on written words in Chinese and English dyslexics despite different activation in Chinese versus English normal readers (Hu et al., 2010).

Given that Chinese children with dyslexia showed noise exclusion deficit, how might it affect reading acquisition? Sperling et al. (2005) proposed three possibilities. (1) The visual impairment is part of a broader problem with noise exclusion that affects speech and further influence reading. (2) The deficit directly affects reading through the visual modality. (3) The visual deficit could have detrimental effects on the development of phonological representations and then affect reading acquisition. For Chinese reading, the effects of noise exclusion deficit on reading impairments might be the possibilities of (2) and (3). First, no matter in Chinese or alphabetic language reading, word recognition requires abstracting away from variations in size, font, and style. It may be more difficult if visual processing is hampered by deficits in noise exclusion. Sperling et al. (2005) second, although different from the letter-by-letter phonemic segments in alphabetic languages, the experience with phonetic radicals of Chinese characters also shapes the development of phonological information. If children have difficulties in extracting phonetic information from noisy distractors, phonological presentation would be affected.

### Noise Exclusion and Visual-Spatial Attention

Given that the noise exclusion deficit might be a cross-cultural deficit, what is the nature of it? In the present study, results showed that dyslexic children exhibited poor contrast sensitivities and coherent sensitivities than controls only in the high-noise condition. It might reveal that for children with dyslexia, the distractors were more difficult to inhibit. Researchers proposed that signal enhancement and noise exclusion (inhibition of distractors) are two mechanisms of visual-spatial attention to optimize perceptual judgment. Noise exclusion can help to improve the perceptual filtering so that signals are processed and noise is excluded (Sperling et al., 2006). However, the invalid attention window of DD during processing will expose the target stimuli to the spatial noisy distractors (Facoetti et al., 2008). Therefore, some researchers believe that the noise exclusion deficit shown in DD is essentially caused by visual-spatial attention deficit (Facoetti et al., 2008). Previous studies have shown the attention impairments of dyslexia: individuals with dyslexia cannot shift their attention from one window to another and have a prolonged attentional dwell time, suggesting their sluggish attentional shifting (Hari and Renvall, 2001). Effective attention shifting plays an important role in reading. However, people with dyslexia exhibited spatial sluggish attentional shifting (e.g., Ruffino et al., 2010b, 2014; Vidyasagar and Pammer, 2010) in visual sense modalities (Facoetti et al., 2010), which might finally lead to the poor reading performance. Some studies found that visual selective attention deficits in dyslexia may be due to a specific difficulty in orienting and focusing and a diffused distribution of visual processing resources (Facoetti et al., 2000a, b). Other studies also found that the noise exclusion deficit in DD could be moderated by visual-spatial attention (Ruffino et al., 2010a; Conlon et al., 2012).

### The Relationship Between Noise Exclusion Hypothesis and Magnocellular Theory

Even though the findings of this study supported noise exclusion hypothesis, it still cannot exclusively rule out magnocellular theory. The magnocellular theory was a neural physiological interpretation of visual deficits in dyslexia, but the noise exclusion hypothesis was described as a behavioral level theory. In actuality, the noise exclusion might also have its underlying neural mechanism. As mentioned above, it is undeniable that perceptual noise exclusion is closely related to visual-spatial attention. In the frontoparietal network, the parietal posterior cortex (PPC) is one of the essential areas for visual spatial attention (Saalmann et al., 2007). It seems that the noise exclusion is related to the function of PPC. In addition, PPC is also considered as a part of the magnocellular-dorsal pathway (Saalmann et al., 2007). Therefore, researchers argued that noise exclusion might be related to the magnocellular-dorsal pathway. Because of the large visual receptive field and the fast conduction velocity, the magnocellular system provides an initial rapid, low-spatial-frequency signal, possibly through the dorsal stream to the parietal and frontal regions (Vidyasagar, 2005). This early activation is thought to provide an initial global analysis of the object foreground/background segregation, before feedback signals into the inferotemporal cortex fill in the details (Laycock, 2012). This indicates that the magnocellular-dorsal pathway theory and the noise exclusion hypothesis are not two completely opposite theoretical hypothesis, especially in the brain network. We believe they may reflect DD’s dysfunction in different levels of the visual system. The magnocellular-dorsal theory may emphasize the atypical functional characteristics of different stages of the visual conduction pathway in dyslexia, especially the early stages, while the noise exclusion hypothesis may emphasize the abnormal top-down regulation by the high-order cortex of early visual processing in dyslexia. This hypothesis should be examined in future research using a brain imaging method.

## Limitations

There were several limitations to this study. First, we only used a “low achievement” criterion to screen the Chinese children with dyslexia (reading ability score below −1.5 SD), but it was not a mainstream in an international context. We should use the “persistence” and/or “resistance” criterion to screen dyslexia strictly in the future. Second, the participants in two experiments were not the same group of children. We will further test the reliability of the results in the same group of children in the future. Third, in experiment 1, the Gabor contrast sensitivity task manipulated the contrast ratio of stimuli. It cannot probe the function of M or P pathway strictly, because the M pathway was sensitive to the stimuli with high temporal frequency, low spatial frequency and low contrast, and the P pathway was sensitive to the stimuli with low temporal frequency, high spatial frequency and high contrast. Future research should design a better paradigm to more strictly detect the function of M and P pathway.

## Data Availability Statement

The data that support the findings of this study are available from the corresponding author upon reasonable request.

## Ethics Statement

The studies involving human participants were reviewed and approved by the Institute of Psychology, Chinese Academy of Sciences. Written informed consent from the participants’ legal guardian/next of kin was not required to participate in this study in accordance with the national legislation and the institutional requirements.

## Author Contributions

YJ and H-YB designed the study. YJ conducted the experiments, analyzed the data, wrote and revised the manuscript with the help of H-YB.

## Conflict of Interest

The authors declare that the research was conducted in the absence of any commercial or financial relationships that could be construed as a potential conflict of interest.
